# Stable and Thin-Polymer-Based Modification of Neurovascular Stents with 2-Methacryloyloxyethyl Phosphorylcholine Polymer for Antithrombogenicity

**DOI:** 10.3390/bioengineering11080833

**Published:** 2024-08-15

**Authors:** Naoki Inuzuka, Yasuhiro Shobayashi, Satoshi Tateshima, Yuya Sato, Yoshio Ohba, Kazuhiko Ishihara, Yuji Teramura

**Affiliations:** 1 R&D Department, Japan Medical Device Startup Incubation Program, 3-7-2 Nihonbashihon-cho, Chuo-ku, Tokyo 103-0023, Japan; naoki.inuzuka@jmprogram.org; 2 R&D Department, N.B. Medical Inc., 3-7-2 Nihonbashihon-cho, Chuo-ku, Tokyo 103-0023, Japan; 3 Division of Interventional Neuroradiology, Department of Radiological Sciences, David Geffen School of Medicine, University of California Los Angeles (UCLA), Ronald Reagan UCLA Medical Center, 757 Westwood Plaza, Suite 2129, Los Angeles, CA 90095, USA; 4 Department of Bioengineering, Graduate School of Engineering, The University of Tokyo, 7-3-1 Hongo, Bunkyo-ku, Tokyo 113-8656, Japan; 5 Cellular and Molecular Biotechnology Research Institute (CMB), National Institute of Advanced Industrial Science and Technology (AIST), AIST Tsukuba Central 5, 1-1-1 Higashi, Ibaraki, Tsukuba 305-8565, Japan; ooba.y@aist.go.jp; 6 Division of Materials & Manufacturing Science, Graduate School of Engineering, Osaka University, 2-1 Yamadaoka, Suita, Osaka 565-0871, Japan; 7 Department of Immunology, Genetics and Pathology (IGP), Uppsala University, Dag Hammarskjölds väg 20, 751 85 Uppsala, Sweden; 8 Master’s/Doctoral Program in Life Science Innovation (T-LSI), University of Tsukuba, 1-1-1 Tennodai, Ibaraki, Tsukuba 305-8577, Japan

**Keywords:** neurovascular stent, intracranial aneurysm, anti-thrombotic coating, 2-methacryloyloxyethyl phosphorylcholine polymer, surface modification, silane coupling

## Abstract

The advent of intracranial stents has revolutionized the endovascular treatment of cerebral aneurysms. The utilization of stents has rendered numerous cerebral aneurysm amenable to endovascular treatment, thereby obviating the need for otherwise invasive open surgical options. Stent placement has become a mainstream approach because of its safety and efficacy. However, further improvements are required for clinically approved devices to avoid the frequent occurrence of thrombotic complications. Therefore, controlling the thrombotic complications associated with the use of devices is of significant importance. Our group has developed a unique stent coated with a 2-methacryloyloxyethyl phosphorylcholine (MPC)-based polymer. In this study, the surface characteristics of the polymer coating were verified using X-ray photoelectron spectroscopy and atomic force microscopy. Subsequently, the antithrombotic properties of the coating were evaluated by measuring platelet count and thrombin–antithrombin complex levels of whole human blood after 3 h of incubation in a Chandler loop model. Scanning electron microscopy was utilized to examine thrombus formation on the stent surface. We observed that MPC polymer-coated stents significantly reduced thrombus formation as compared to bare stents and several clinically approved devices. Finally, the coated stents were further analyzed by implanting them in the internal thoracic arteries of pigs. Angiographic imaging and histopathological examinations that were performed one week after implantation revealed that the vascular lumen was well maintained and coated stents were integrated within the vascular endothelium without inducing adverse effects. Thus, we demonstrated the efficacy of MPC polymer coating as a viable strategy for avoiding the thrombotic risks associated with neurovascular stents.

## 1. Introduction

Subarachnoid hemorrhage (SAH) is a critical condition with poor prognosis and high mortality rates ranging from 32 to 67% [1]; moreover, approximately 30% of SAH survivors experience permanent disabilities that impair their daily activities [2]. Because SAH is predominantly caused by the rupture of cerebral aneurysms, the treatment of incidental cerebral aneurysms is pivotal for preventing SAH [3]. Multiple therapeutic modalities have been developed to treat cerebral aneurysms, including conventional open surgical treatment and minimally invasive endovascular treatment. The advent of the coil embolization technique in early 1990s resulted in a paradigm shift to endovascular treatment. Consequently, approximately 70% of cerebral aneurysms are treated with endovascular coiling, while fewer aneurysms are treated with open surgical clipping [4,5]. Although coil embolization has gained popularity because of its high efficacy and safety across various clinical studies [6], certain limitations have been reported [7]. For example, coiling is sufficient for the embolization of aneurysms with a narrow neck; however, larger aneurysm with wide-necked configuration cannot be treated using only coils. To overcome this limitation, coil-assisted stents that prevent coils from dropping into the parent vessel have been developed. The use of coil-assisted stents for the treatment of aneurysms has been well established, with numerous studies demonstrating their efficacy [8]. This broader application of stents implies lower recurrence rates of aneurysms when using coil-assisted stents than when using coiling alone, demonstrating the versatility and effectiveness of stents. Thus, a wide range of aneurysm sizes and types can be treated with coil-assisted stents [9].

However, the increased use of metal implants in cerebral arteries has inherently resulted in a higher rate of thrombotic complications. In clinical practice, thrombotic complications associated with different stent implantations have been reported (e.g., 3.3–4.7% for Neuroform Atlas^®^ (Stryker, Fremont, CA, USA) [10,11] and 8.7–14.3% for Enterprise^®^ (Johnson and Johnson, Irvine, CA, USA) [12,13]). Metallic parts are required to support the integrity of the coils and counter the effects of flow diversion to ensure treatment efficacy [14]. Thus, optimization of stent design, combined with adequate surface treatment to mitigate the risk of thrombotic complication, should be entertained. For instance, heparin coating is used for the Gore Viabahn^®^ stent (W. L. Gore & Associates, Inc., Newark, NJ, USA) to enhance its antithrombotic properties for peripheral stenting [15]. Surfaces covered with the phosphorylcholine (PC) group, a representative phospholipid polar group, exhibit superior blood compatibility, such as resistance to both thrombus formation and complement activation. An accumulation of PC groups is employed in the Pipeline™ Flex Embolization Device with Shield Technology™ (Medtronic, Irvine, CA, USA), which has been clinically demonstrated to be effective in reducing thrombotic events [16]. As a PC-bearing polymer, 2-methacryloyloxyethyl phosphorylcholine (MPC) polymers have been used for various medical devices [17,18]. These examples highlight the significance of surface treatments in improving the safety and efficacy of stents for endovascular therapy. Various methods of modifying the surface of metallic stents have been studied to improve hemocompatibility; however, the effect of surface modification strongly depends on the stent design and coating methods because the exposed metallic area and blood flow on each stent are quite different.

Our group originally developed a coil-assisted stent for cerebral aneurysms, featuring a completely novel design that combines the characteristics of both open- and closed-cell designs. This hybrid structure combines the flexibility and conformability of open-cell stents with the high structural strength and resheathability of closed-cell stents. However, the developed stent exhibits a risk of thrombosis, owing to its unique structure.

In this study, we investigated the application of MPC polymer coatings on coil-assisted stents to improve their antithrombotic properties. Here, we used the Chandler loop system with whole human blood to study the hemocompatibility of a coated stent by comparing its thrombotic responses with those of an uncoated bare stent, the U.S. Food and Drug Administration (FDA)-approved Neuroform Atlas^®^ Stent System, and the Enterprise^®^ stent by measuring platelet counts and coagulation markers. Moreover, we evaluated the thrombotic score based on scanning electron microscopy (SEM) analysis of the stents after blood testing. Finally, we implanted the coated stent into a porcine internal thoracic artery for one week to evaluate its in vivo safety and study its endothelialization through histopathological examination.

## 2. Materials and Methods

### 2.1. Coating of Stent and Flat Metallic Plate with 2-Methacryloyloxyethyl Phosphorylcholine Polymer

We constructed a stent with a self-expanding design (diameter: 4.0 mm; length: 15 mm) by performing laser cutting of an Ni–Ti alloy tube and subsequent heat treatment to activate its shape-memory characteristics, followed by electropolishing to achieve a smooth surface finish. This process was conducted in house and termed NBM-SES-01. To modify the stent using the MPC polymer, poly(MPC-*co*-3-trimethoxysilylpropyl methacrylate) (Lipidure^®^ 3001, NOF CORPORATION, Tokyo, Japan), we followed the procedure provided by the company with a minor modification. The MPC polymer was dissolved in ethanol (4.0 wt%) and mixed with an equal volume of 0.10 mM hydrochloric acid under continuous stirring for 30 min at room temperature. For the surface treatment, the stent was subjected to ultraviolet–ozone irradiation for 15 min using a generator (SKB401Y, SUN ENERGY Co., Ltd., Tokyo, Japan) before being submerged in the MPC polymer solution for 1 min. Then, the treated stent was dried at 80 °C for 1 h and subsequently washed with distilled water to thoroughly remove unbound polymers. We also applied the same procedure to coat a flat Ni–Ti alloy plate with the same composition as the stent.

### 2.2. Surface Characterization

#### 2.2.1. X-ray Photoelectron Spectroscopy

X-ray photoelectron spectroscopy (XPS; KRATOS ULTRA2, Shimadzu Co., Kyoto, Japan) was used to characterize the surface composition of the coated stents. Precise measurements were ensured by setting the take-off angle of the photoelectron to 90° and maintaining the operating pressure below 1.0 × 10^−7^ Pa. Calibration of binding energies was conducted using the carbon 1s (C_1_s) peak at 285.0 keV, which is commonly attributed to alkyl groups. Elemental analyses of the stent surfaces were performed by quantifying the peak areas corresponding to each element. XPS analysis was conducted on six distinct areas of each stent (*n* = 3).

#### 2.2.2. Atomic Force Microscopic Observation

Morphological analysis of the coated stent surface was performed via atomic force microscopy (AFM), which was performed by the Research Institute of Biomolecule Metrology Co., Ltd. (Tsukuba, Japan), using their AFM microscope. We measured the morphology and thickness of the MPC polymer coating on the flat Ni–Ti alloy plate in phosphate-buffered saline (PBS) via AFM. The observations were performed in different areas (dimensions of 10 µm × 10 µm). The thickness of the polymer layer was measured by peeling it off using an ultra-short cantilever (USC-F1.5-k0.6, Nanoworld, Neuchâtel, Switzerland) and measuring the step difference from the Ni–Ti alloy surface.

### 2.3. Blood Compatibility Assay Using Chandler Loop Model

To evaluate the blood compatibility of the coated stent, we used the Chandler loop model with a polyurethane tube (60 cm in length, with an internal diameter of 4.0 mm) and plastic connector, which were coated with one of the following MPC polymers: poly(MPC-*co*-*n*-butyl methacrylate (BMA)) or Lipidure^®^ CM5206 (NOF CORPORATION, Tokyo, Japan), which was previously established in our laboratory [19]. The tube was filled with a 0.5% Lipidure^®^ CM5206 ethanol solution, then rotated at 10 rpm for 3 h at room temperature. After thoroughly removing the excess solution, the tube was completely air-dried. In the blood experiment, we carefully inserted a coated or bare stent into the treated tube and positioned it at the end located opposite to the connector. We collected fresh whole human blood from healthy volunteers in Venoject II vacuum blood collecting systems (Terumo Co., Tokyo, Japan) under the approved JMPR-EMPR-220607-01 protocol and immediately added heparin solution to achieve a final concentration of 1.0 IU/mL, ensuring the absence of air bubbles. Then, we poured the whole blood (2 mL) into a tube with a stent and sealed both ends to form a closed loop, which was mounted on a rotating platform in an incubator. The assembly was rotated at 10 rpm for 3 h at 37 °C. After incubation, the blood and stent were collected separately for analysis. The blood was immediately mixed with ethylenediaminetetraacetic acid (Thermo Fisher Scientific Inc., Grand Island, NY, USA). The collected stent was thoroughly rinsed with physiological saline and stored in a 4% phosphate-buffered paraformaldehyde (PFA) solution. The platelet count was measured using a pocH80i hematology analyzer (Sysmex Corporation, Kobe, Japan), in which the initial platelet count from each donor was considered as the baseline to calculate the platelet consumption percentage after 3 h of incubation. The blood was centrifuged (2500× *g*, 10 min, 4 °C) to separate the plasma, which was then stored at −80 °C. To analyze a representative coagulation marker, the thrombin–antithrombin complex (TAT) concentration in plasma was determined using an enzyme-linked immunosorbent assay kit (ASSAYPRO LLC, Saint Charles, MI, USA), and the investigation was performed directly on undiluted plasma samples.

### 2.4. Scanning Electron Microscopic Observation

We washed the stents, which were stored in PFA solution with physiological saline and pure water, then air-dried them for SEM observations using a TM4000PlusII microscope (Hitachi High-Tech, Tokyo, Japan). For a comprehensive assessment, each stent was evaluated using images obtained at 20 randomly selected locations. The images were then subjected to a semi-quantitative evaluation based on the specific criteria listed in Table 1, as adopted from [20].

### 2.5. In Vivo Implantation of Coated Stents in Pigs

Coated and bare stents were sterilized with ethylene oxide gas and then utilized for in vivo experiments using healthy male Göttingen minipigs aged 11–18 months and weighing 20–30 kg. All porcine-based experiments were conducted at the Shuzenji Branch of Hashima Laboratory, Nihon Bioresearch Inc. These experiments adhered to the Basic Guidelines for Animal Experimentation proposed by the Ministry of Health, Labour, and Welfare of Japan. The animal experiments were approved by the Animal Experiment Committee of the facility. Previously quarantined and acclimated pigs were individually housed in stainless-steel cages under controlled conditions (16–27 °C, 12 h light–dark cycle) and administered clopidogrel (75 mg/body) and aspirin (200 mg/body) via mixed feed from three days before stent placement until necropsy. Stents were placed in the internal thoracic artery (which is commonly used in minipig stent experiments) of two male pigs because the diameters of these arteries were appropriate for stent placement. Preoperative sedation was achieved through the intramuscular administration of a mixture of atropine sulfate, medetomidine hydrochloride, and midazolam. Heparin was administered before the procedure (3 mL/head), and the activated clotting time was measured using Actalyke MINI II, with additional heparin doses being administered intravenously every 1–2 h. Postoperative care involved waking the pigs with atipamezole hydrochloride and supporting them with oxygen until they were fully awake, along with the administration of ampicillin sodium for three days and buprenorphine hydrochloride for pain management. Seven days after implantation, a guiding catheter was inserted to capture angiographic images (ARCADIS GEN 2, Siemens, Germany) to assess the vascular condition. Following necropsy, we performed a histopathological evaluation of the vessels with the stents. During necropsy, deep anesthesia was induced via an intravenous injection of thiopental sodium, followed by euthanasia through exsanguination of the abdominal aorta, ensuring humane treatment of the animals.

### 2.6. Statistical Analysis

GraphPad Prism 10 (GraphPad Software, San Diego, CA, USA) was used for one-way analysis of variance, followed by Tukey’s multiple comparison test for post hoc analysis, enabling the evaluation of statistical significance across the experimental data.

## 3. Results and Discussion

### 3.1. Surface Characterization

We conducted XPS analyses of the MPC polymer-coated and bare stents to study their surface elemental compositions (Table 2). P, C, N, and O atoms, which are constituents of MPC polymer, were detected in the coated stents. In particular, P was strongly detected on the coated stents (6.42 ± 1.04%), whereas no P was detected on the bare stent surface. This result indicates that the surface of the Ni–Ti alloy was coated with the MPC polymer. In addition, we checked the stability of the polymer coating after we stored the coated stent for 8 months (Supplementary Table S1). No significant changes in the compositions were observed, indicating that the coating remains stable over a long time.

**Figure 1:**
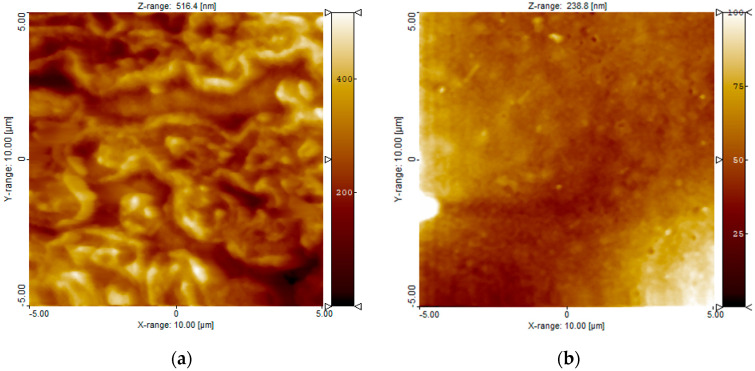
Representative atomic force microscopic image of an Ni-Ti flat substrate coated with 2-methacryloyloxyethyl phosphorylcholine (MPC) polymer. (**a**) Bare Ni-Ti flat substrate; (**b**) MPC polymer-coated Ni-Ti flat substrate. Swelled MPC polymer was detected over the entire surface in the measurements conducted in phosphate-buffered saline.

We also used AFM for surface characterization of the MPC polymer-coated surface. The AFM observations were conducted in PBS, in which the state of the MPC polymer was similar to that in blood (Figure 1). AFM images revealed that the entire surface was equally coated with the MPC polymer; moreover, the thickness of the hydrated MPC polymer layer was 35.6 ± 3.6 nm, on average, which was analyzed by the scratching of the MPC polymer layer from the bare surface. Thus, both XPS and AFM data showed the presence of MPC polymer on the Ni–Ti alloy surface, with the MPC polymer immobilized on the surface. It is widely known that a silane coupling can be formed via covalent bonding and used for the immobilization of polymers via SiOH groups [21,22]. Therefore, we considered the MPC polymer to be covalently immobilized on the surface.

**Figure 2:**
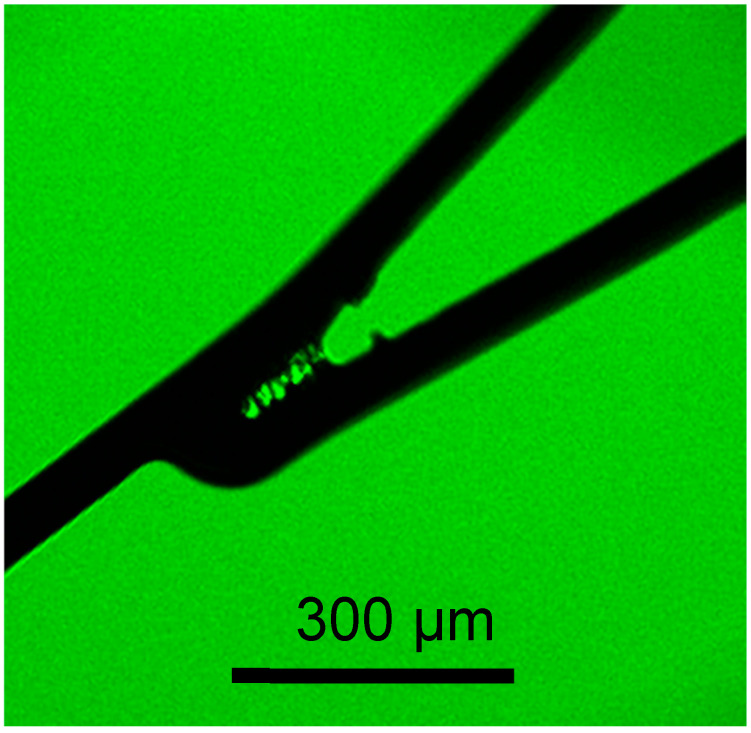
MPC polymer deposition between narrow struts after the coating with Lipidure^®^ CM5206 via hydrophobic interaction. Visible MPC polymer can be observed between the narrow struts, in which the deposition forms bridges after air drying of the polymer solution. Such deposition can lead to the potential risk of distal embolism in neurovascular applications.

Several methods are used to immobilize MPC polymers on substrate surfaces [18,23]. One is hydrophobic interaction-based immobilization using a copolymer with hydrophobic monomer units, such as BMA (Lipidure^®^ CM5206 is a water-insoluble polymer, owing to both the high composition of the hydrophobic unit and its high molecular weight). The polymer forms a hydrated layer on the substrate surface in an aqueous medium [18]. This approach has been used in numerous clinical devices, such as artificial hearts [24]. However, this type of immobilization is difficult to apply to stents. As shown in Figure 2, a visible MPC polymer was deposited between the narrow struts, forming insoluble bridges or polymer films after the polymer solution was air-dried. We were concerned with the coating stability after the washing process. Therefore, we attempted to optimize the washing process to remove residual polymer; however, completely removing the polymer between the struts was impossible; consequently, a small amount of polymer deposition remained visible. Such defects are unacceptable for neurovascular stents because they increase the risk of embolic events. Moreover, stress testing of the stents under conditions simulating clinical use revealed that particles over 70 μm were generated from the coated stents. This issue is not acceptable for neurovascular applications, where no particles larger than 70 μm are acceptable [25].

Therefore, we concluded that this hydrophobic interaction-based coating is inappropriate for use in neurovascular stents. In contrast, we employed a chemical reaction with a silane coupling reagent to coating the MPC polymer on stents (Supplementary Figure S1). With this chemical bonding method, we were able to completely wash out unbound polymers from the stent surface, as shown in Figure 2. Consequently, no particulate formation was observed under stress-testing conditions similar to those applied for the Lipidure^®^ CM5206-coated stent. Notably, the 70 µm or larger particles observed in clinical simulation tests with the Lipidure^®^ CM5206-coated stents did not occur in our samples. The absence of residual particles in this stent indicates the stability of the coating. This is a significant advantage, especially for stents used in the microvasculature of the brain, where particle formation can lead to severe complications, such as distal embolism. Thus, we chose silane coupling to coat the stent with the MPC polymer to ensure the stability of the polymer.

### 3.2. In Vitro Evaluation: Whole-Blood Test Using Chandler Loop

**Figure 3:**
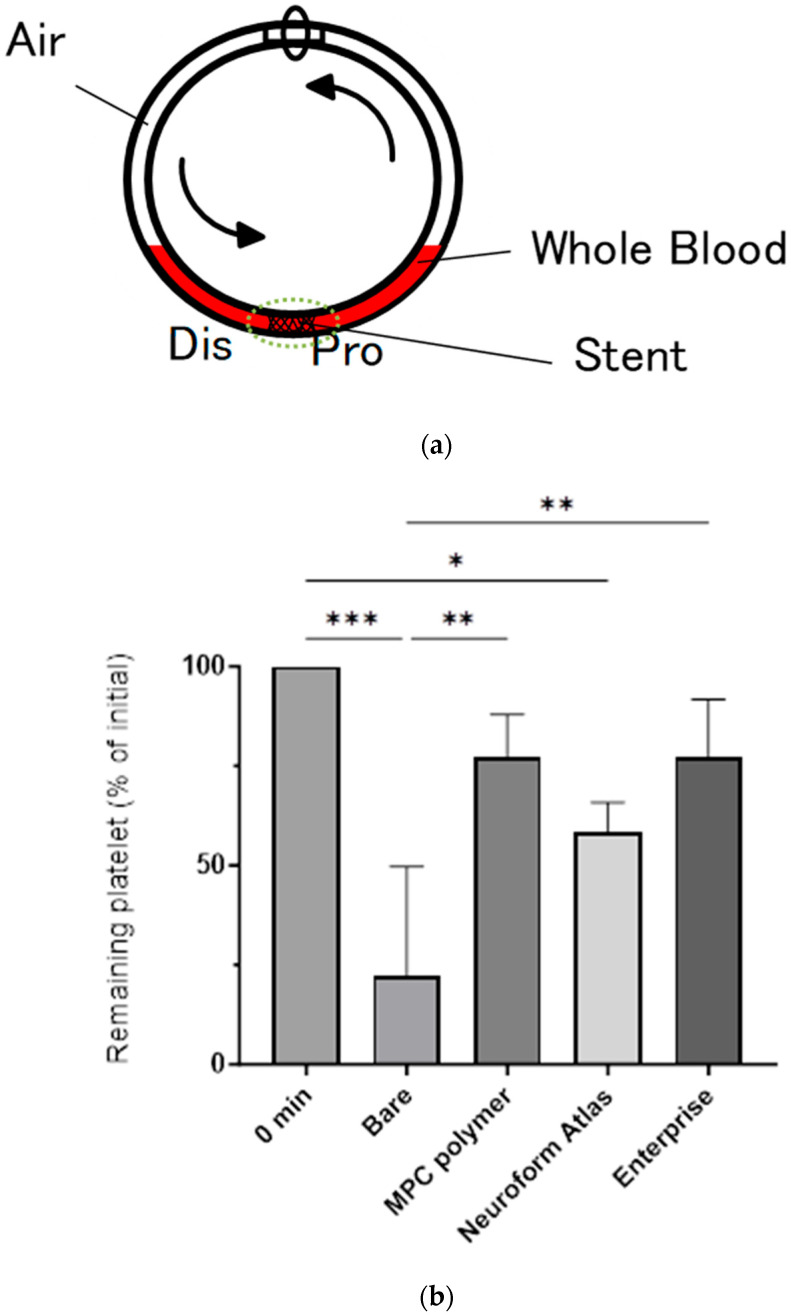

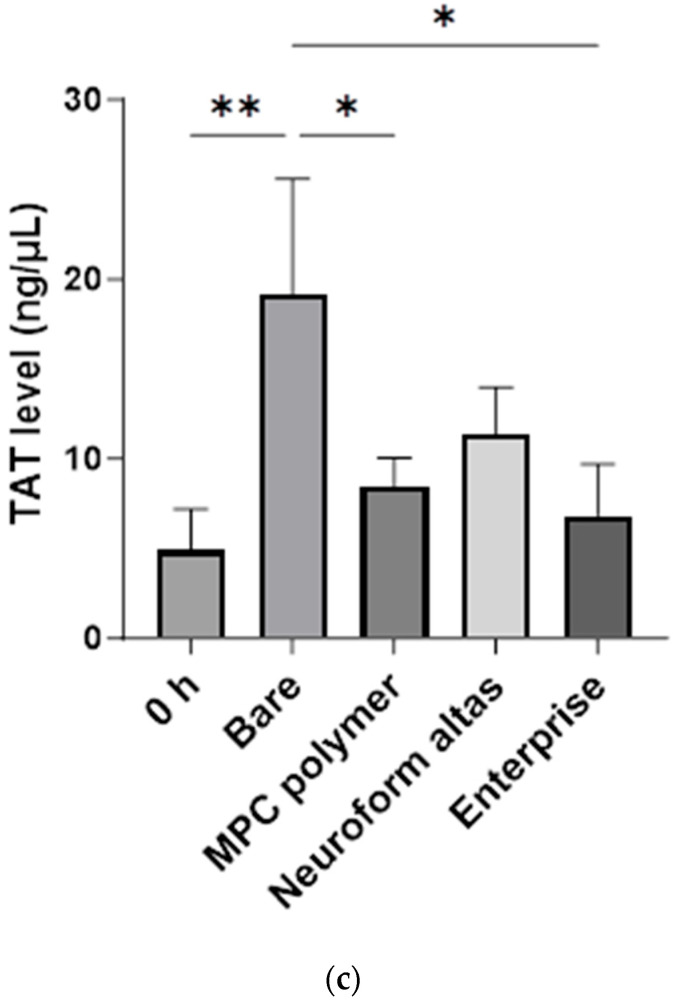
Blood compatibility test of stents using the Chandler loop model with whole human blood. (**a**) Schematic illustration of the Chandler loop model. In this model, the stent is placed opposite the connector in the tube, and fresh whole blood (2 mL) is poured into the tube, while the remaining volume is filled with air. Here, a bare stent, MPC polymer-coated stent, Neuroform Atlas^®^, and Enterprise^®^ were tested. (**b**) Relative platelet counts in the whole blood following the blood experiment using the Chandler loop model. The initial platelet count was set as the baseline (100%) when blood sampling was performed immediately after the commencement of the test. The figure shows the percentage of platelets remaining in the blood after 3 h of contact with the stent. Each stent showed a statistically significant difference compared to the bare stent (*** *p* < 0.005, ** *p* < 0.01, * *p* < 0.05). (**c**) Relative thrombin–antithrombin complex (TAT) concentration in the blood after 3 h of incubation. The initial TAT concentration was set as the baseline (100%), which was measured on blood samples collected immediately after the commencement of the test. Blood samples from three different experiments (*n* = 3) were analyzed, and the data are expressed as the mean percentage ± standard deviation. The stent coated with MPC polymer significantly suppressed the increase in TAT compared to the bare stent (** *p* < 0.01, * *p* < 0.05).

We conducted in vitro whole-blood testing using the Chandler loop by inserting either a bare or an MPC polymer-coated stent (Figure 3a). For comparison, we used the Neuroform Atlas^®^ and Enterprise^®^ stents, which are commonly utilized in clinical settings. Neuroform Atlas^®^ features an open-cell design, whereas Enterprise^®^ is composed of closed cells. Both stents, which have no antithrombotic coating, have shown high safety and efficacy profiles in clinical practice. During whole-blood testing, the stents were continuously exposed to whole blood for 3 h, followed by the measurement of platelet counts (Figure 3b) and TAT levels as coagulation markers (Figure 3c). The remaining number of platelets in the blood after circulation in the loop containing the MPC polymer-coated stents was 77 ± 11%, whereas it was 22 ± 28% for bare stents. This indicates that platelet aggregation was significantly suppressed by the MPC polymer coating. As a positive control, the remaining numbers of platelets were determined to be 58 ± 8 and 77 ± 14% when using Neuroform Atlas^®^ and Enterprise^®^, respectively, indicating that the performance of the controls was equivalent to that of the MPC polymer-coated stents. We also compared the TAT levels among the samples and determined that the TAT concentration for the bare stent (19.2 ± 6.4 ng/mL) was significantly higher than the concentrations in other groups (8.5 ± 1.6 ng/mL for the MPC polymer-coated stent, 11.4 ± 2.6 ng/mL for Neuroform Atlas^®^, and 6.8 ± 2.9 ng/mL for Enterprise^®^, with the control at 0 h showing a concentration of 4.9 ± 2.3 ng/mL). This indicates that the coagulation system was more activated by the contact of blood with the bare stent surface than that with the coated stent and controls.

**Figure 4:**
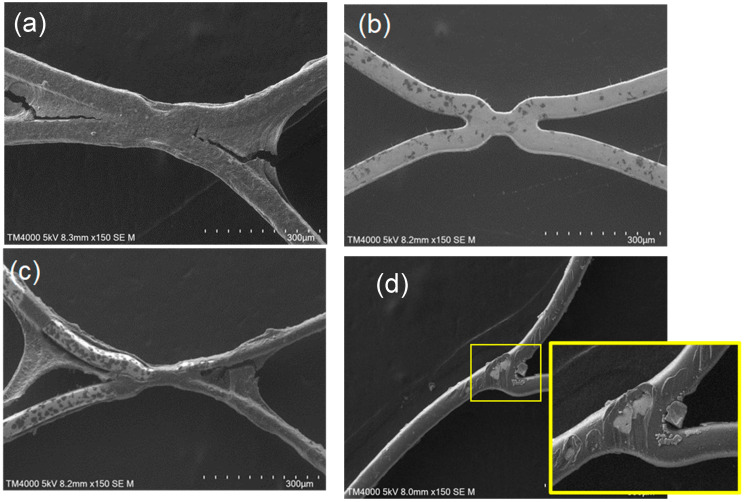
Representative scanning electron microscopic (SEM) images of four different stent samples at 3 h in the Chandler loop model. (**a**) Bare stent; (**b**) MPC polymer-coated stent; (**c**) Neuroform Atlas^®^; (**d**) Enterprise^®^. The yellow box is an image that shows a 3x magnification of the central part of the image.

**Figure 5:**
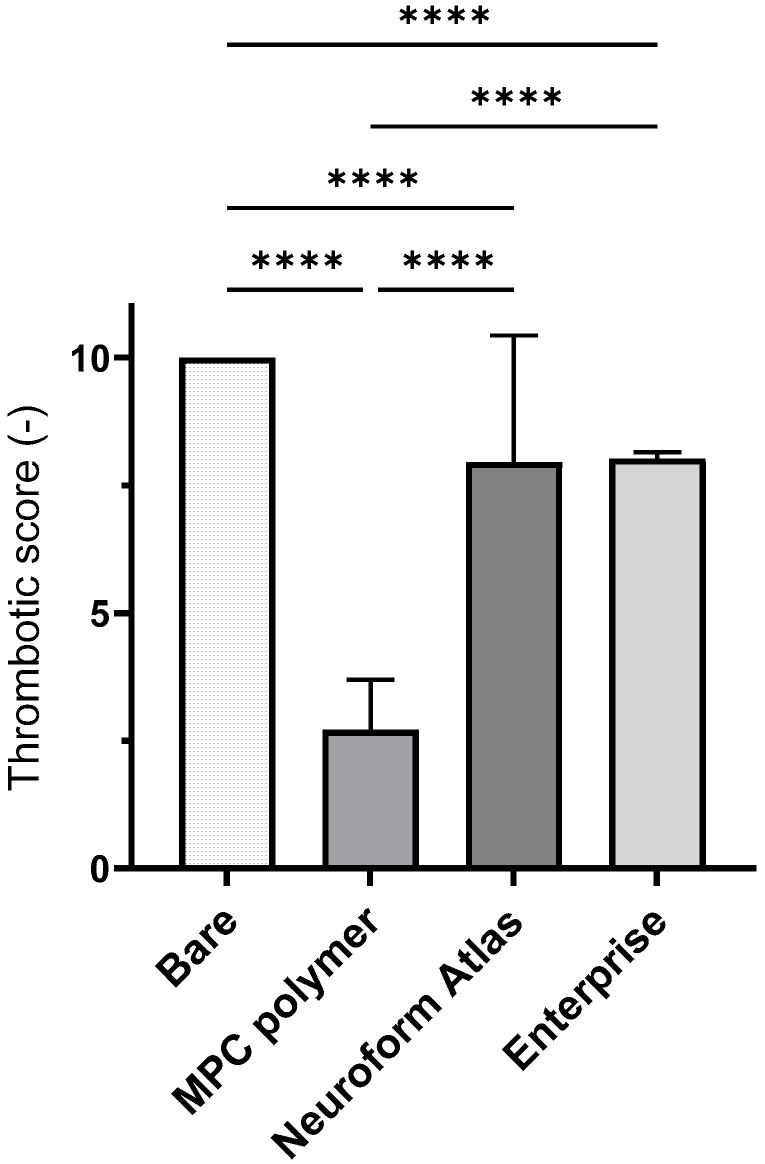
Thrombogenicity scoring of stents based on analysis. For each stent, SEM images were captured at 20 predefined locations, ensuring comprehensive coverage. Scores were assigned to each stent at these 20 distinct locations based on the criteria established in Table 1. MPC polymer-coated surfaces had significantly reduced thrombogenicity scores compared to other stents, indicating improved antithrombotic performance (**** *p* < 0.001).

We also analyzed the stent surfaces via SEM, with 20 different sites randomly selected for observation (Figure 4a: bare stent; Figure 4b: MPC polymer-coated stent; Figure 4c: Neuroform Atlas^®^; Figure 4d: Enterprise^®^). Thick and layered thrombus formation was observed on the bare stent, whereas almost no thrombus formation occurred on the MPC polymer-coated stent, although a small clot was formed on the surface. Thick thrombus formation was detected on the Neuroform Atlas^®^, similar to the case of the bare stent. Conversely, thick thrombus formations were not detected on Enterprise^®^; however, small thrombus formation was observed over the entire surface. We then evaluated the thrombus formation scores of all stents based on the criteria outlined in Table 1 (Figure 5). Bare stents exhibited a thrombus score of 10.0 ± 0.2, indicating a significantly higher level of thrombus formation as compared to that on other stents. The Neuroform Atlas^®^, Enterprise^®^, and MPC polymer-coated stent had scores of 8.0 ± 2.5, 8.0 ± 0.1, and 2.7 ± 1.0, respectively. Thus, the amount of thrombus formation on MPC polymer-coated stents was significantly lower than that on other FDA-approved stents, indicating the higher efficacy of the MPC polymer coating in reducing thrombus formation on coated stents.

### 3.3. In Vivo Evaluation: Stent Placement in Internal Thoracic Artery of a Pig

**Figure 6:**
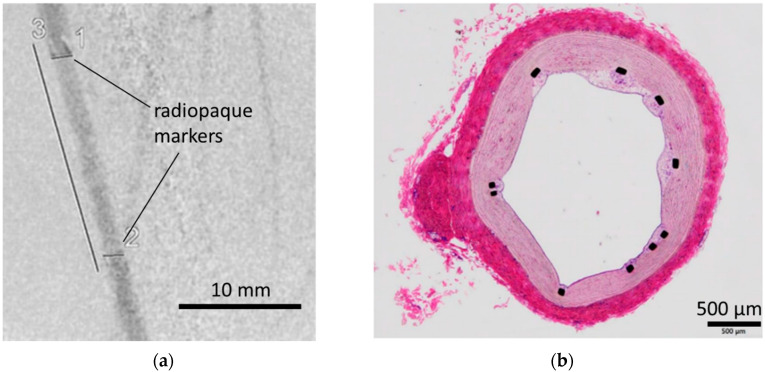
In vivo evaluation of an MPC polymer-coated stent that was implanted in the internal thoracic artery of a pig. (**a**) Representative angiographic image 1 week after stent implantation. The numbers in the figure represent the respective measurement points: 1 indicates the proximal stent diameter, 2 indicates the distal stent diameter, and 3 indicates the stent length. The corresponding measurements are as follows: 1 = 1.8 mm, 2 = 1.8 mm, 3 = 19 mm.; (**b**) representative image of hematoxylin and eosin staining of a sliced section from the stent-implanted vessels. Black dots represent struts of stents.

For in vivo evaluation of safety issues, we placed MPC polymer-coated stents in the internal thoracic artery of a pig for a week, followed by the assessment of vascular conditions via angiography, necropsy, and histopathological examination (Figure 6). The angiographic images showed that the vascular lumen was well preserved, and no visible clots occurred during the one-week period post implantation (Figure 6a). The general health of the recipients showed no abnormalities, indicating that acute safety issues were not a cause for concern with respect to MPC polymer-coated stents. Moreover, histopathological examination showed no thrombus formation; samples were assessed in different sections (Figure 6b). We calculated the percentage of struts that were covered by tissue to be 87.5 ± 14.4%, indicating that almost all struts were covered by neointimal tissue within a week and no inhibition of tissue growth was observed. Thus, we observed that neointimal tissue was not inhibited when the stent structure was coated with the MPC polymer. The MPC polymer coating is inert to the immunological system, owing to its strong antifouling properties, and tissue growth occurs without inflammation to cover the coated struts [18]. It is noted that this large animal study is limited by the small sample size (*n* = 2). The presented data analysis was based on an observational study, and no statistical analysis was conducted. However, we will perform further analysis with larger number of pig studies.

Coating with synthetic polymers exhibiting antifouling properties, such as MPC polymers, has been demonstrated to be effective for antithrombogenicity in various clinical studies [26,27,28,29]. In this study, we investigated the MPC polymer coating of our stent using the NBM-SES-01 design for blood compatibility. Our stent exhibited thick thrombus formation when compared FDA-approved stents; however, coating this stent with the MPC polymer reduced thrombus formation. Initial blood contact is crucial for thrombus formation with platelets and the fibrin network [30], and it is influenced by several factors, including the presence of atherosclerosis and other pathological conditions, as well as the degree of adhesion and potential damage to the vascular wall caused by stents [31,32]. These complexities underscore the challenge of identifying the specific causes of stent thrombosis. However, our study showed that the MPC polymer coating of stents could significantly reduce the risk of thrombus formation. For coil-assisted stents used in aneurysm treatment, crucial aspects such as coil support ability and flow diversion effect depend on the stent design, including its shape and surface area. An increase in the surface area of a stent can reduce the blood flow into the aneurysm, enhancing the therapeutic effect [14]. However, a stent design intended to enhance stent performance might inadvertently increase the risk of thrombus formation, owing to the increased surface area, which can be a tradeoff between improving stent efficacy and the associated risk of thrombosis. An increase in surface area not only contributes to better treatment outcomes by limiting blood entry into an aneurysm but also spontaneously elevates the risk of thrombus formation on the surface of the stent. Therefore, MPC polymer coatings can potentially mitigate the thrombotic risks associated with increased surface area without reducing therapeutic efficacy. Furthermore, this result is important because the coating allows for the potential design of stents with a larger total surface area. A previous study shows that the greater the contact area with the vessel, the stronger the expansion force [33]. This advantage is particularly significant in cases requiring strong expansion forces, such as atherosclerotic lesions, where adopting a stent design with a larger surface area can be beneficial. Although MPC polymer coating is known to inhibit in vitro cell adhesion, our findings indicated that, seven days after vascular implantation, approximately 90% of the stent struts were well-covered with an endothelial layer. Because the MPC polymer is intertwined with biological systems, the coating did not hamper the growth of the neointimal tissue, resulting in full coverage of the stent. According to a report by Lewis et al., endothelial cells that surround stents can bridge thin structures, owing to the inert properties of MPC polymer [34].

## 4. Conclusions

We coated our designed stents with an MPC polymer via covalent silane coupling and observed that no platelet adhesion or fibrin formation occurred on the treated surface, which was placed in whole human blood. Because thick thrombus formation was detected on the bare stent surface in the blood, MPC polymer coating via covalent silane coupling was effective in preventing the contact activation of blood. Moreover, no detached particles were detected in the coated stent during stress testing, indicating the stability of the polymer coating. An in vivo study on stent placement in the internal thoracic artery of pigs demonstrated that the vascular lumen was well preserved, and no visible clots were detected for a week post implantation. Moreover, the general health conditions of the recipients were normal, indicating the high level of safety of the coated stent. Owing to the high biocompatibility of the MPC polymer-coated surface, the growth of the neointimal tissue was not inhibited on the coated surface, and the struts of the stents were well covered by new tissue. Thus, MPC polymer coating is a promising approach for reducing thrombotic risks in neurovascular stents.

## Figures and Tables

**Table 1:** Evaluation criteria of thrombotic scores.

Score	Evaluation Criteria
Level 1	Almost no thrombus formation on the stent surface, which remains flat.
Level 2	Slight thrombus formation on the stent surface, but it is mostly flat.
Level 3	Thin thrombus formation observed in localized areas of the stent surface.
Level 4	Wide areas of the stent surface covered with a thin thrombus, but overall, it is flat.
Level 5	Stent surface is predominantly flat, but localized, raised thrombi are observed.
Level 6	Wide areas of the stent surface are covered with a thin thrombus, with localized, raised thrombi.
Level 7	Stent surface is predominantly covered with a thin thrombus, with some areas having raised thrombi.
Level 8	Entire stent surface is covered with thrombi, with numerous localized, raised thrombi.
Level 9	Entire stent surface is covered with thick, raised thrombi, with some areas having significantly thick, multilayered thrombi.
Level 10	Entire stent surface is completely covered with significantly thick, multilayered thrombi.

**Table 2:** Atomic composition percentages on the stent surface, as quantified using X-ray photoelectron spectroscopy.

Stent Sample		Atomic Component (%)						
		P(2p)	Si(2p)	C(1s)	N(1s)	Ti(2p)	O(1s)	Ni(2p3/2)
Bare	Mean	0.0	0.0	35.5	0.6	27.6	32.3	3.3
(*n* = 6)	SD	0.0	0.0	12.6	0.7	9.9	3.8	2.1
MPC polymer-coated	Mean	4.4	0.1	46.3	1.2	13.3	32.2	0.9
(*n* = 6)	SD	1.3	0.2	11.6	0.3	6.2	4.9	1.4

## Data Availability

The raw data supporting the conclusions of this article will be made available by the authors on request.

## Supplementary Materials

The following supporting information can be down-loaded at: https://www.mdpi.com/article/10.3390/bioengineering11080833/s1, Figure S1: Schematic illustration of MPC polymer immobiliza-tion on Ni-Ti surface; Table S1: Atomic composition percentages on the 8 months aged stent surface.
