# Supplementary material for: Stable and Thin-Polymer-Based Modification of Neurovascular Stents with 2-Methacryloyloxyethyl Phosphorylcholine Polymer for Antithrombogenicity
Source: Bioengineering (Basel). 2024 Aug 15;11(8):833. doi: 10.3390/bioengineering11080833 (PMC11351483; doi:10.3390/bioengineering11080833)
Supplement: Supplementary file 1 [file bioengineering-11-00833-s001.zip › bioengineering-3113758-Supplementary materials.pdf]

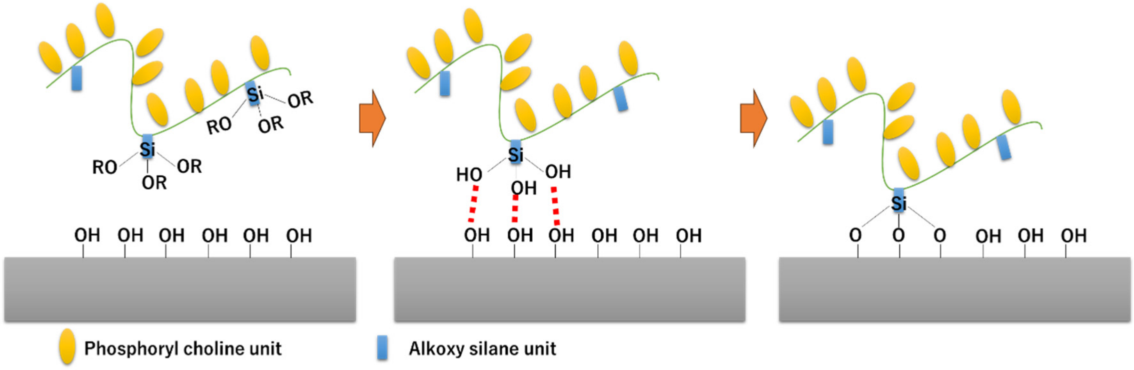

Supplementary Figure S1. Schematic illustration of MPC polymer immobilization on Ni-Ti surface.

Supplementary Table S1. Atomic composition percentages on the 8 months aged stent surface, as quantified using X-ray photoelectron spectroscopy

Stent sample		Atomic component (%)					
		P(2p)	C(1s)	N(1s)	Ti(2p)	O(1s)	Ni(2p3/2)
MPC polymer-coated (n=6)	Mean	7.1	35.8	1.8	18.7	36.7	0.0
	SD	1.6	6.5	1.2	7.9	1.3	0.0
